# *Helicobacter suis* infection is associated with nodular gastritis‐like appearance of gastric mucosa‐associated lymphoid tissue lymphoma

**DOI:** 10.1002/cam4.2314

**Published:** 2019-06-18

**Authors:** Hidehiko Takigawa, Satoshi Masaki, Toshikatsu Naito, Ryo Yuge, Yuji Urabe, Shinji Tanaka, Kazuhiro Sentani, Taiji Matsuo, Keisuke Matsuo, Kazuaki Chayama, Yasuhiko Kitadai

**Affiliations:** ^1^ Department of Gastroenterology and Metabolism Hiroshima University Hiroshima Japan; ^2^ Department of Health and Science Prefectural University of Hiroshima Hiroshima Japan; ^3^ Department of Endoscopy Hiroshima University Hiroshima Japan; ^4^ Department of Molecular Pathology Hiroshima University Hiroshima Japan; ^5^ Department of Internal Medicine Matsuonaika Hospital Mihara Japan

**Keywords:** eradication therapy, *Helicobacter suis*, mucosa‐associated lymphoid tissue, nodular gastritis, non‐*H pylori Helicobacter* (NHPH)

## Abstract

Most patients with gastric mucosa‐associated lymphoid tissue (MALT) lymphoma are infected with *Helicobacter pylori*, and eradication therapy is the first‐line treatment for localized disease with *H pylori* infection. However, there were several reports showing effectiveness of eradication therapy in even *H pylori* negative cases. Gastric MALT lymphomas are endoscopically classified into three common types: superficial, ulcerative, and elevated types. For the past 20 years, we have encountered 200 cases of localized gastric MALT lymphoma. Among them, only 4 cases (2%) showed similar macroscopic findings to those of nodular gastritis (gastric MALT lymphoma with nodular gastritis‐like appearance; M‐NGA). Here, we compared clinicopathological characteristics and prevalence of non‐*H pylori Helicobacter* (NHPH) infection between M‐NGA and other common types of gastric MALT lymphoma. To examine the prevalence of NHPH infection, DNA was extracted from formalin‐fixed paraffin‐embedded biopsy tissues from four cases of M‐NGA, 20 cases of common endoscopic types of gastric MALT lymphoma, and 10 cases of nodular gastritis. We used a highly sensitive polymerase chain reaction assay to detect the presence of five species of NHPH (*Helicobacter suis*, *H felis*, *H bizzozeronii*, *H salomonis*, and *H heilmannii*). *H suis* infection was detected in 4, 2, and 0 of the 4, 20, and 10 cases of M‐NGA, other types of gastric MALT lymphoma, and nodular gastritis, respectively. Other NHPH species were not detected in any cases. Complete response rate by eradication therapy was 4/4 in M‐NGA cases. Therefore, nodular gastritis‐like MALT lymphoma, which shows a very rare phenotype, is closely associated with NHPH infection, and eradication therapy may be the first‐choice treatment.

## 1 INTRODUCTION

*Helicobacter pylori (H pylori)* is a major cause of atrophic gastritis and is also involved in the pathogenesis of several gastrointestinal diseases, including gastric ulcer, gastric adenocarcinoma, and gastric mucosa‐associated lymphoid tissue (MALT) lymphoma.^1^ Recently, non‐*H pylori Helicobacters* (NHPHs; also referred to as *H heilmannii*‐like organisms or *H heilmannii* sensu lato) have been found to be associated with a range of gastric disorders, especially MALT lymphoma.^2^, ^3^ Five NHPH species (*H suis*,* H felis*,* H bizzozeronii*,* H salomonis*, and *H heilmannii*) are known to infect humans and cause gastritis.^3^ While NHPH infection is very common in dogs, cats, pigs, and nonhuman primates,^4^, ^5^ its prevalence in humans is much lower than that of *H pylori*, ranging from 0.1% to 6%.^6^, ^7^, ^8^, ^9^, ^10^, ^11^, ^12^, ^13^

Nodular gastritis, a form of chronic gastritis referred to as “gooseflesh‐like gastritis,” is frequently associated with follicular lymphoid hyperplasia with intraepithelial lymphocytosis.^14^ The term “nodular” is not included in the Sydney classification because it is not yet widely accepted by pathologists worldwide. Endoscopically, gastric MALT lymphomas are usually classified into three types: superficial, ulcerative, or elevated types. Gastric MALT lymphoma with nodular gastritis‐like appearance (M‐NGA) is very rare.^15^ In the present study, we examined the prevalence of NHPH infection and its association with macroscopic features of gastric MALT lymphoma.

## 2 PATIENTS AND METHODS

### 2.1 Patients

From 1996 to 2015, 200 patients (79 men and 121 women; median age: 62 years) were diagnosed with gastric MALT lymphoma and followed up at the Hiroshima University Hospital after treatment. We macroscopically classified gastric MALT lymphoma into three types: superficial, elevated, and ulceration types (collectively referred to as the common type MALT lymphoma in this study). Of the 200 cases, 196 were common type MALT lymphoma, and the remaining four cases exhibited a distinctive appearance with a nodular elevation diffusely extending from the antrum to the corpus. This appearance is very similar to that of nodular gastritis. Therefore, the four cases were classified as M‐NGA. In this study, we randomly selected 20 cases from the 196 cases of common type MALT lymphoma and then compared clinicopathological data and status of NHPH infection between common type MALT lymphoma and M‐NGA. The cases were examined for *H pylori* infection and the presence of the *API2‐MALT1* chimeric transcript. *H pylori* infection was evaluated serologically by anti‐*H pylori* IgG antibody and by the urea breath test. In the more recent cases, polymerase chain reaction (PCR) was performed to assess *IGH* gene rearrangements using Invivoscribe IGH chain clonality assay (Invivoscribe Technologies, San Diego, CA) according to the manufacturer's instructions. The presence of gastric mucosal atrophy was determined by endoscopic examination based on the criteria of Kimura and Takemoto as previously described.^16^ All patients were endoscopically followed every 6 months. MALT lymphoma was determined to exhibit complete response (CR) when the absence of lymphoma was confirmed both macroscopically and pathologically. The characteristics, treatments, and outcomes of the patients were analyzed retrospectively.

To eradicate *Helicobacter* species, patients with gastric MALT lymphoma received a 1‐week course of orally administered lansoprazole (30 mg/day), amoxicillin (750 mg/day), and clarithromycin (400 mg/day). A second endoscopy was performed at 3‐12 months after completion of the antimicrobial treatment. These studies were conducted in accordance with the Declaration of Helsinki and were approved by the Institutional Review Board of the Hiroshima University Hospital. Informed consent was obtained from all patients.

### 2.2 DNA extraction and gastric *Helicobacter* species‐specific PCR assay

Paraffin block sections (10 × 10 μm) were collected in microtubes, and DNA was extracted using the Gene Read DNA FFPE Kit (Qiagen Japan, Tokyo, Japan) following the manufacturer's instructions. PCR amplification of the urease gene from NHPH species (*H suis*,* H bizzozeronii*,* H felis*,* H salomonis*, and *H heilmannii *ss) was performed using species‐specific primers according to a previous report (Table 1).^17^ PCR reactions were conducted using the buffer and DNA polymerase from KOD FX Neo (TOYOBO, Osaka, Japan) according to the manufacturer's instructions. The PCR amplification of *H suis*,* H bizzozeronii*,* H felis*, and *H heilmannii* was performed under the following conditions: 5 minutes of preincubation at 95°C, followed by 40 cycles of 30 seconds at 94°C, 30 seconds at 60°C, and 30 seconds at 72°C. A final extension was performed for 7 minutes at 72°C *H salomonis* amplification was performed with 5 minutes of preincubation at 95°C, followed by 40 cycles of 30 seconds at 94°C, 30 seconds at 62°C, and 30 seconds at 72°C. A final extension was performed for 7 minutes at 72°C as previously reported.^17^ To confirm the specificity of NHPH species‐specific PCR assays, approximately 30% of the positive PCR products was randomly selected for sequencing using both forward and reverse primers. Nucleotide sequences were analyzed using the BLAST tool on the website of the DNA Data Bank of Japan (http://www.ddbj.nig.ac.jp/index-j.html).

**Table 1:** Primers for detection of non‐*H pylori Helicobacter*

Target gene	Target species	Primer sequences (F: forward, R: reverse)	Annealing temperature (℃）	Cycle number	Fragment size (bp)
*ureA*	*H suis*	F: CACCACCCCGGGGAAGTGATCTTG	60	40	253
		R: CTACATCAATCAAATGCACGGTTTTTTCTTCG			
*ureA*	*H bizzozeronii*	F: CGCTTTGAACCCGGTGAGAAAA	60	40	172
		R: TATCGCAACCGCAATTCACAACA			
*ureB*	*H felis*	F: TCCCACTACCGGGGATCGTG	60	40	350
		R: CAGCGGTTACAATCAAGCCCTCA			
*ureAB*	*H salomonis*	F: CTTTGGGTCTGTGCCTGCCTG	62	40	219
		R: CATCGCGGATAGTCTTACCGCCT			
*ureA*	*H heilmannii* ss	F: CTTTCTCCTGGTGAAGTGATTCTC	60	40	368
		R: CAGTTGATGGTGCCAAAG			

### 2.3 Statistical analysis

Between‐group differences were evaluated using Student's *t*‐test for quantitative data and chi‐squared test for categorical data. Yates' correction or Fisher's exact test was used as required. All tests were two‐sided, and a *P* value < 0.05 was considered statistically significant. For multiple comparisons, one‐way analysis of variance (one‐way ANOVA) and the Bonferroni post hoc test were used appropriately. All analyses were performed using EZR (Saitama Medical Centre, Jichi Medical University, Saitama, Japan).^18^

## 3 RESULTS

### 3.1 Case reports of M‐NGA

**Figure 1:**
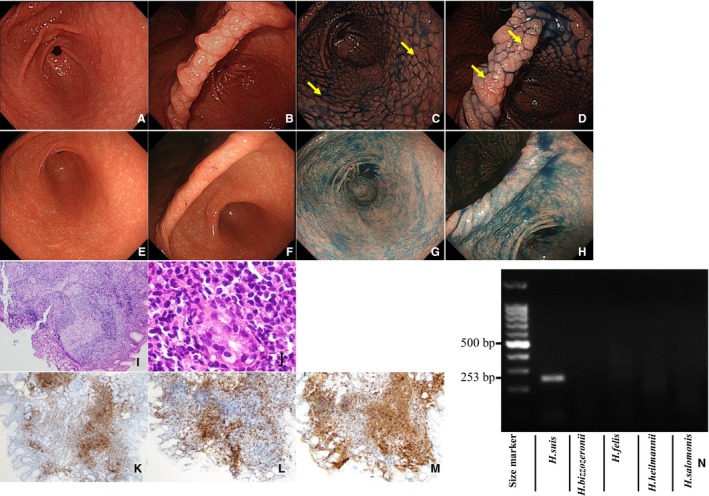
Characterization of case A1. Endoscopic examination (before eradication): Multiple heterogeneous nodules similar to nodular gastritis in antrum (A) and anglus (B). Indigo carmine dye makes the nodules more obvious in the antrum (C) and anglus (D). Endoscopic examination (after eradication): no nodules in the antrum (E) or anglus (F), even with indigo carmine dye in the antrum (G) and anglus (H). Pathological examination (before eradication): Lymphoma cells proliferate diffusely and form heterogeneous nodules with lymphoid hyperplasia. HE, ×100 (I). Atypical lymphoid cells invading the epithelium, resulting in the destruction of mucosal glands and formation of lymphoepithelial lesions. HE, ×400 (J). Immunohistochemistry (before eradication): Neoplastic cells are positive for CD20 (K), negative for CD3 (L), and positive for CD79a (M). PCR assay (before eradication): Positive for *Helicobacter suis* (253 bp); negative for other non‐*H pylori Helicobacter* (N). Biopsies taken from areas indicated with yellow arrows showed pathological findings compatible with mucosa‐associated lymphoid tissue lymphoma. Abbreviation: HE, hematoxylin and eosin

**Figure 2:**
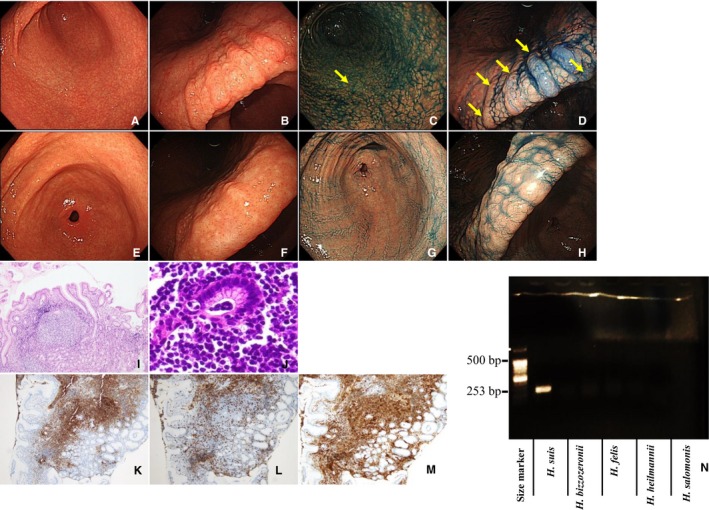
Characterization of case A2. Endoscopic examination (before eradication): Multiple heterogeneous nodules similar to nodular gastritis in antrum (A) and anglus (B). Indigo carmine dye makes the nodules more obvious in the antrum (C) and anglus (D). Endoscopic examination (after eradication): no nodules in the antrum (E) or anglus (F), even with indigo carmine dye in the antrum (G) and anglus (H). Pathological examination (before eradication): lymphoma cells proliferate diffusely and form heterogeneous nodules with lymphoid hyperplasia. HE, ×100 (I). Atypical lymphoid cells invading the epithelium, resulting in the destruction of mucosal glands and formation of lymphoepithelial lesions. HE, ×400 (J). Immunohistochemistry (before eradication): Neoplastic cells are positive for CD20 (K), negative for CD3 (L), and positive for CD79a (M). PCR assay (before eradication): Positive for *Helicobacter suis* (253 bp); negative for other non‐*H pylori Helicobacter* (N). Biopsies taken from areas indicated with yellow arrows showed pathological findings compatible with mucosa‐associated lymphoid tissue lymphoma. Abbreviation: HE, hematoxylin and eosin

**Figure 3:**
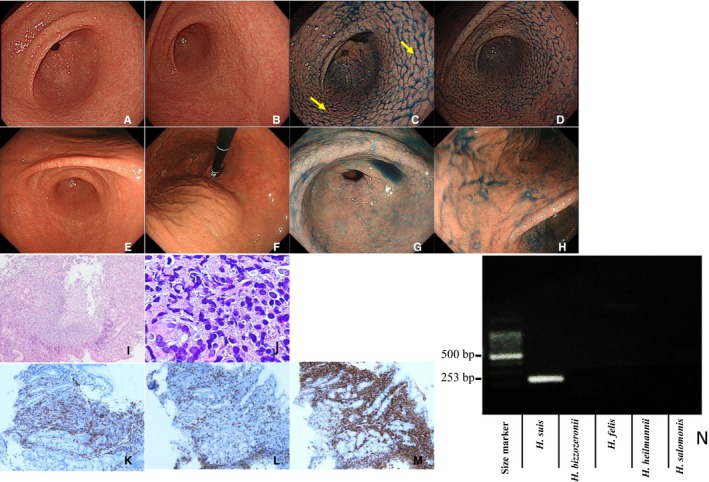
Characterization of case A3. Endoscopic examination (before eradication): Multiple heterogeneous nodules similar to nodular gastritis in antrum (A) and anglus (B). Indigo carmine dye makes the nodules more obvious in the antrum (C) and anglus (D). Endoscopic examination (after eradication): no nodules in the antrum (E) or anglus (F), even with indigo carmine dye in the antrum (G) and anglus (H). Pathological examination (before eradication): lymphoma cells proliferate diffusely and form heterogeneous nodules with lymphoid hyperplasia. HE, ×100 (I). Atypical lymphoid cells invading the epithelium, resulting in the destruction of mucosal glands and formation of lymphoepithelial lesions. HE, ×400 (J). Immunohistochemistry (before eradication): Neoplastic cells are positive for CD20 (K), negative for CD3 (L), and positive for CD79a (M). PCR assay (before eradication): Positive for *Helicobacter suis* (253 bp); negative for other non‐*H pylori Helicobacter* (N). Biopsies taken from areas indicated with yellow arrows showed pathological findings compatible with mucosa‐associated lymphoid tissue lymphoma. Abbreviation: HE, hematoxylin and eosin

**Figure 4:**
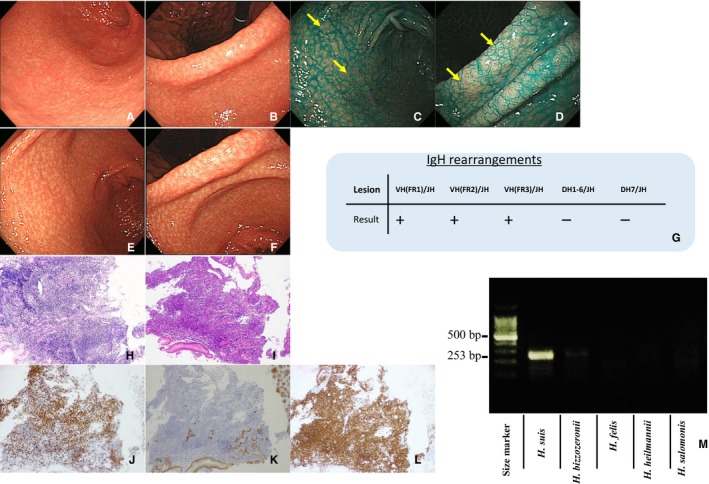
Characterization of case A4. Endoscopic examination (before eradication): Multiple heterogeneous nodules similar to nodular gastritis in antrum (A) and anglus (B). Indigo carmine dye makes the nodules more obvious in the antrum (C) and anglus (D). Endoscopic examination (after eradication): No nodules in the antrum (E) or anglus (F). Status of monoclonal IgH rearrangement (G). Pathological examination (before eradication): Lymphoma cells proliferate diffusely and form heterogeneous nodules with lymphoid hyperplasia. HE, ×100 (H). Atypical lymphoid cells invading the epithelium, resulting in the destruction of mucosal glands and formation of lymphoepithelial lesions. HE, ×400 (I). Immunohistochemistry (before eradication): Neoplastic cells are positive for CD20 (J), negative for CD3 (K), and positive for CD79a (L). PCR assay (before eradication): Positive for *Helicobacter suis* (253 bp); negative for other non‐*H pylori Helicobacter* (M). Biopsies taken from areas indicated with yellow arrows showed pathological findings compatible with mucosa‐associated lymphoid tissue lymphoma. Abbreviation: HE, hematoxylin and eosin

Clinicopathological characteristics of the four cases of M‐NGA are shown in Table 2 (case A1‐A4). All four patients were males, with an average age of 40 years. Representative endoscopic and pathological images and PCR assay results for case A1 are shown in Figure 1. Endoscopically, the gastric mucosa exhibited a coarse, granular appearance in the antrum, angles, and lower corpus (Figure 1A,B). Indigo carmine was used to enhance and image the nodules (Figure 1C,D). Pathological findings compatible with MALT lymphoma were detected in biopsy tissues taken from areas indicated with yellow arrows in Figure 1C,D. Nodular size was larger and more heterogeneous as compared to that of typical nodular gastritis. Histologically, a diffuse distribution of small lymphocytes with slightly irregular nuclei was observed (Figure 1I). Lymphoid cells proliferated diffusely and formed nodules of various sizes with follicular hyperplasia (Figure 1I). The atypical lymphoid cells had invaded the epithelium, resulting in the destruction of mucosal glands and formation of lymphoepithelial lesions (Figure 1J). Neoplastic cells were positive for CD20 (Figure 1K), negative for CD3 (Figure 1L), and positive for CD79a (Figure 1M). *H suis* infection was confirmed by PCR (Figure 1N). The endoscopic and pathological images of the other three cases (A2, A3, and A4) are shown in Figures 2, 3, 4. Endoscopic and pathological features were similar in all four cases of M‐NGA (Figures 1A‐D,I‐M; 2A‐D,I‐M; 3A‐D,I‐M; and 4A‐D,H‐L). *IGH* rearrangement was evaluated in case A4, and monoclonal IgH rearrangement was confirmed (Figure 4G). Serum anti‐*H pylori* IgG antibody and urea breath tests were only positive in case A3. The other three cases were negative for *H pylori* infection. Although *H suis*‐specific PCR band was detected in all cases, pathological diagnosis was very difficult. An NHPH with a straight appearance, corkscrew‐shaped spirals, and large size was pathologically observed in proper gastric gland regions with hematoxylin and eosin staining only in case 2. Three months after eradication therapy with lansoprazole, amoxicillin, and clarithromycin, we confirmed that *H suis* infection had disappeared by PCR. Moreover, at 3 months after eradication therapy, gastroscopy showed that the coarse, granular appearance and enlarged mucosal folds were no longer present, and multiple heterogeneous whitish spots were observed in all four cases (Figures 1E‐H, 2E‐G, 3E‐G, and 4E,F). CR was confirmed pathologically.

**Table 2:** Cases of gastric MALT lymphoma with nodular gastritis‐like appearance

No.	Age (y)	Sex	HP	API2‐MALT1	Treatment	Eradication regimen	Outcome	Gastric mucosa atrophy	Bacterial species (PCR)	NHPH diagnosis (histological)	NHPH after eradication	Authors
A1	36	M	N	N	Eradication	LAC	CR	(‐)	*H suis*	N	N	Current study
A2	37	M	N	N	Eradication	LAC	CR	(‐)	*H suis*	P	N	
A3	42	M	P	N	Eradication	LAC	CR	C‐2	*H suis*	N	N	
A4	45	M	N	N	Eradication	LAC	CR	(‐)	*H suis*	N	N	
B1	ND	ND	N	ND	Eradication	LAC	CR	ND	ND	P	N	Okiyama et al^10^
B2	ND	ND	N	ND	Eradication	LAC	CR	ND	ND	P	N	Okiyama et al^10^

Abbreviations: CR, complete response; F, female; HP, *H pylori* infection; LAC, lansoprazole, amoxicillin, clarithromycin; M, male; MALT, mucosa‐associated lymphoid tissue; N, negative; ND, not described; NHPH, non‐*H pylori Helicobacter*; P, positive.

### 3.2 Prevalence of NHPH infection in M‐NGA

**Figure 5:**
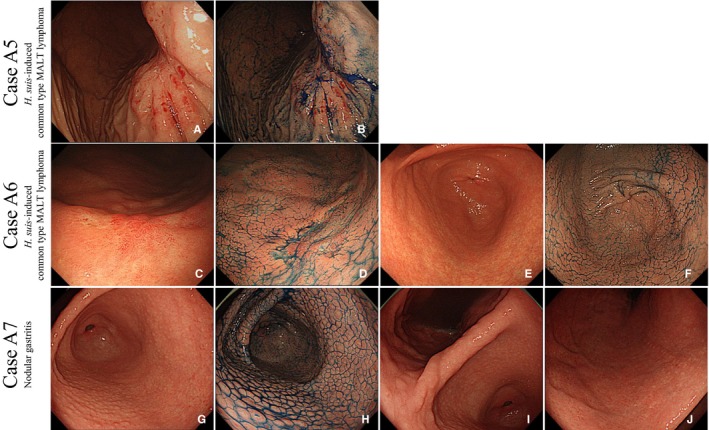
Characterization of cases A5, A6, and A7. Cases A5 and A6: Endoscopic pictures of common type MALT lymphoma with *Helicobacter suis* infection. In case A5, the superficial phenotype of gastric MALT lymphoma was observed in the gastric body under white light (A) and chromoendoscopy (B). In case A6, the superficial phenotype was observed in the gastric body under white light (C) and chromoendoscopy (D). Mild nodular gastritis without MALT lymphoma was observed in the gastric antrum under white light (E) and chromoendoscopy (F). Case A7: Representative endoscopic pictures of nodular gastritis. Endoscopic examination (before eradication): Multiple homogeneous nodules are seen in the antrum. Whitish spots exist on the top of each nodule (G). No nodule is observed in the gastric anglus (H). Mild atrophy is observed in the gastric body (I). Indigo carmine dye makes the nodules more obvious in the antrum (J). Abbreviation: MALT, mucosa‐associated lymphoid tissue

To compare the prevalence of NHPH infection, we utilized 20 cases of common type gastric MALT lymphoma for which the clinical course had been followed for more than 2 years. Notably, all four cases of M‐NGA were infected with *H suis*, which is one of the NHPH; however, *H pylori* infection was detected in only one case (case A3). In common type MALT lymphoma, the prevalence of *H pylori* infection was 75% and that of NHPH was 10%, indicating that the prevalence rate of NHPH infection was significantly higher in M‐NGA than in common type MALT lymphoma (Table 3). In two cases of common type MALT lymphoma, *H suis* infection was detected, both of which had superficial type MALT lymphoma. No characteristic findings such as nodules were found in the areas of MALT lymphoma in either case (Figure 5E‐H). However, in case A6, an endoscopic finding similar to nodular gastritis was observed in the antrum area (Figure 5E,5). MALT lymphoma was pathologically detected only in superficial lesions observed in the gastric body, not in this antrum lesion.

**Table 3:** Clinicopathological features of M‐NGA, common type gastric MALT lymphoma, and nodular gastritis

	M‐NGA (n = 4)	Common type (n = 20) $\left(\begin{array}{c} \text{Superficial}\;17\\ \text{Ulceration}\;2\\ \text{Elevated}\;1 \end{array}\right)$	Nodular gastritis (n = 10)	*P*‐value $\left(\begin{array}{c} \text{M - NGA vs Common type}\\ \text{Common type vs Nodular gastritis}\\ \text{Nodular gastritis vs M - NGA} \end{array}\right)$
Age: average ± SD	40 ± 3.6	64 ± 12.6	38 ± 13	0.0012* 0.00011* 0.7427
Sex: male/female	4/0 (100%)	10/10 (50%)	7/3 (70%)	0.064 0.2974 0.22
API2‐MALT1 chimeric transcript: positive/negative	0/4 (0%)	1/19 (5%)	ND	0.6478 ND ND
HP infection: positive/negative	1/3 (25%)	15/5 (75%)	10/0 (100%)	0.053 0.083 0.002*
NHPH infection: positive/negative	4/0 (100%)	2/18 (10%)	0/10 (10%)	0.0001* 0.30 0.0002*
Effect of eradication therapy: CR/NC	4/0 (100%)	13/7 (65%)	10/0 (100%)	0.16 0.033 1

Abbreviations: CR, complete response; HP, *Helicobacter pylori*; M‐NGA, gastric MALT lymphoma with nodular gastritis‐like appearance; MALT, mucosa‐associated lymphoid tissue; NC, no change; ND, no data; NHPH, non‐*H pylori Helicobacter*.

* Statistically significant (significance level = 0.05/3).

Ten cases of nodular gastritis were also examined, all of which were positive for *H pylori* infection but negative for NHPH infection. Among these three groups, sex and efficacy of eradication therapy were not significantly different. Average age was significantly lower in M‐NGA and nodular gastritis groups than in the common type MALT lymphoma group. *H pylori* infection rate was significantly higher in the nodular gastritis group than in the M‐NGA group. Remarkably, infection rate of NHPH was significantly higher in M‐NGA group than in the other two groups.

## 4 DISCUSSION

In this paper, we reported four cases of M‐NGA, focusing on the relationship between M‐NGA and NHPH infection. According to previous reports, NHPH species are more likely to induce MALT lymphoma than other *Helicobacter* species.^19^, ^20^, ^21^ There are several methods to detect NHPHs, including culture, immunohistochemistry, rapid urease test, urea breath test, serum antibody, and stool antigen; however, their low sensitivity limits their application for diagnosis.^22^, ^23^, ^24^ Genetic diagnosis using PCR is the most effective method for diagnosing NHPH infections.^25^ In all of our M‐NGA cases, infection of *H suis*, one of the NHPH species, was detected by PCR analysis. Among them, one case exhibited double infection with *H pylori* and *H suis*, while the other three cases were negative for *H pylori* infection.

It has been reported that *H suis* can result in benign phenotypes, such as gastritis or gastric ulcers, and that it causes relatively mild changes compared with those of *H pylori* infection.^25^, ^26^, ^27^, ^28^ In terms of the general histological features of *H suis*, it has been reported that *H suis* infection causes lymphoid hyperplasia, epithelial hyperplasia, and parietal cell necrosis.^25^, ^26^, ^27^, ^28^ *H suis* infection has been reported to induce lymphoid hyperplasia in animal experiments; this lymphoid hyperplasia occurs in the surface mucosa but not the deep mucosa, and these changes are known to be observed mainly in the L region of the stomach.^28^, ^29^ *H suis* is also reported to cause epithelial cell hyperproliferation.^30^ Furthermore, NHPHs have induced mucosal hypertrophy/nodular hyperplasia in experimental models.^31^ In general, the more inflammation present in the mucosa, the stronger the mononuclear cell infiltration and neutrophil infiltration observed. We speculate that these phenomena could cause an endoscopic nodular gastritis‐like appearance and that hyperplastic stimulation could subsequently cause malignant lymphoma. Unlike *H pylori*, the duration of *H suis* infection could be transient.^32^ Thus, NHPHs may induce endoscopic changes in a relatively short period of time. In contrast, the prevalence of NHPH infection in the common type gastric MALT lymphoma was significantly lower than that in M‐NGA (4/4, 100% vs 2/18, 16%) (Table 3). We observed two cases (cases A5 and A6) of *H suis*‐induced common type MALT lymphoma. In both cases, the phenotype of MALT lymphoma was the superficial type (Figure 5A‐D), and while nodular gastritis was observed in the antrum of one case (Figure 5E,5), MALT lymphoma was not detected in the lesion. From these cases, it can be inferred that *H suis* infection does not necessarily exhibit a nodular gastritis‐like appearance. In nodular gastritis, all cases were infected with *H pylori*, but not NHPH.

We also summarized the clinicopathological characteristics of six cases of M‐NGA (Table 2), which include three cases reported previously (B1‐B2)^10^ and our four identified cases (A1‐A4). The median age of all patients was 40 years. All six cases had NHPH infection, but only one patient was co‐infected with *H pylori*. In all six cases, NHPH infection was eradicated, and MALT lymphoma cells completely disappeared following eradication therapy comprising PPI + clarithromycin + amoxicillin for 1 week. Atrophic gastritis was observed in only one case with *H pylori* and *H suis* infections (case A3). In the other M‐NGA lymphoma cases, endoscopic and pathologic examinations did not reveal any atrophic change in the gastric mucosa. This is consistent with previous reports that showed that NHPH is less likely to cause atrophic gastritis.^25^, ^32^, ^33^

We also compared endoscopic findings between nodular gastritis and M‐NGA. Endoscopically, nodular gastritis shows diffuse and homogenous miliary pattern that is localized to the antrum. Typical endoscopic pictures are shown in Figure 5A‐D (case A5). Homogeneous nodules having whitish spots on the top were observed in the gastric antrum (Figure 5A,B), but not in the gastric anglus (Figure 5C). Mild atrophy was observed in the gastric body (Figure 5D) different from the observation in the M‐NGA cases. In contrast, M‐NGA nodules were larger and more heterogeneous and extended to the corpus of the stomach. Atrophic change in the gastric mucosa was detected in nodular gastritis but not in M‐NGA. It is of considerable interest to know the lateral spread of MALT lymphoma in the endoscopic images, as it is possible that nodular gastritis is the precursor of MALT lymphoma. In all four cases of M‐NGA, biopsies were taken from the area where nodules were observed (Figure 1C,D, 2C,D, 3C, and 4C,D). All tissues taken from the areas showed pathological findings compatible with MALT lymphoma.

In conclusion, we assessed four cases of M‐NGA, a distinct type of gastric MALT lymphoma. M‐NGA is closely associated with NHPH infection. Both NHPH infection and MALT lymphoma cells were completely eradicated by eradication therapy. Therefore, NHPH infection may be one of the causes of the pathogenesis of M‐NGA.

## CONFLICT OF INTEREST

The authors declare that there is no conflict of interest regarding the publication of this paper.

## Data Availability

The data that support the findings of this study are available from the corresponding author upon reasonable request.
